# Thyroid function in Danish greenhouse workers

**DOI:** 10.1186/1476-069X-5-32

**Published:** 2006-12-06

**Authors:** Gunnar Toft, Allan Flyvbjerg, Jens Peter Bonde

**Affiliations:** 1 Department of Occupational Medicine, Aarhus University Hospital, Norrebrogade 44, build 2C, DK-8000 Aarhus C, Denmark; 2 The Medical Research Laboratories, Clinical Institute and Medical Department M (Diabetes and Endocrinology), Aarhus University Hospital, DK-8000 Aarhus C, Denmark

## Abstract

**Background:**

From animal studies it is known that currently used pesticides can disturb thyroid function.

**Methods:**

In the present study we investigated the thyroid function in 122 Danish greenhouse workers, to evaluate if greenhouse workers classified as highly exposed to pesticides experiences altered thyroid levels compared to greenhouse workers with lower exposure. Serum samples from the greenhouse workers were sampled both in the spring and the fall to evaluate if differences in pesticide use between seasons resulted in altered thyroid hormone levels.

**Results:**

We found a moderate reduction of free thyroxine (FT4) (10–16%) among the persons working in greenhouses with a high spraying load both in samples collected in the spring and the fall, but none of the other measured thyroid hormones differed significantly between exposure groups in the cross-sectional comparisons. However, in longitudinal analysis of the individual thyroid hormone level between the spring and the fall, more pronounced differences where found with on average 32% higher thyroid stimulating hormone (TSH) level in the spring compared to the fall and at the same time a 5–9% lower total triiodthyroxin (TT3), free triiodthyroxine (FT3) and FT4. The difference between seasons was not consistently more pronounced in the group classified as high exposure compared to the low exposure groups.

**Conclusion:**

The present study indicates that pesticide exposure among Danish greenhouse workers results in only minor disturbances of thyroid hormone levels.

## Background

Disturbances of the function of the thyroid gland can be caused by a number of natural and synthetic compounds. Pesticides may interfere with thyroid hormone homeostasis through many mechanisms of action, i.e. at the receptor level, in binding to transport proteins, in cellular uptake mechanisms or in modifying the metabolism of thyroid hormones (reviewed in [1]). The effects of pesticides on human thyroid function have not been investigated in detail. A study of Mexican pesticide applicators exposed to ethylenebis(dithiocarbamate) (EBDC) revealed an increase in TSH [2]. Similarly, among pesticide formulators in India TSH was increased and TT3 was reduced [3]. Among American pesticide applicators from the Red River Valley of Minnesota, the level of TSH and FT4 decreased from the summer to the fall (after the main spraying season was over) [4]. Except for the decrease in FT4 in the latter study, these findings can be explained by an effect on thyroid function at the peripheral level causing a decreased level of circulating thyroid hormone and consequently an increase of TSH by negative feedback mechanisms. Also animal studies of thyroid disturbances after pesticide exposure that indicate that hypothyroid effect are the general effects, but the effects varies somewhat depending on the specific exposure [5].

Pesticide exposure of greenhouse workers has been determined to be at a level where health risks may occur [6], and especially dermal exposure after reentry to a pesticide treated greenhouse, with manual handling of plants is associated with high transfer of pesticides [7].

In addition to estimating the current exposure, cumulative lifetime exposure to pesticides may be an important determinant of possible adverse health effects. Therefore, in the present study we hypothesize that current and/or cumulative lifetime exposure of greenhouse workers may be related to thyroid disturbances, which mainly should be seen as hypothyroid effects among the most highly exposed workers. Furthermore seasonal differences in pesticide use may cause differences in individual thyroid hormone levels, if the effects are reversible. This will be evaluated in longitudinal analysis of individual thyroid hormones between seasons.

## Materials and methods

### Recruitment and data collection

The participants were recruited by contacting greenhouse owners of 38 greenhouses with 3 or more employees in the autumn of 1993. Thirty-four of the greenhouse owners permitted a meeting for all male workers at their workplace, where we informed about the project. We invited all 199 male workers who fulfilled the inclusion criteria (age 18–45 years, normal puberty, no vasectomy, no known azoospermia, no malignant disease, and no intake of sulfasalazine, β-adrenergic blocking drugs or androgens) to sign-up for the study. A total of 122 men (61%) agreed to participate. None of the men included in the study had known thyroid disease or diabetes. The characteristic of the study population is described in Table 1.

**Table 1:** Characteristics of the study population 122 greenhouse workers given as mean (SD) for continuous covariates and % of the population in the presented category for dichotomous covariates.

**Characteristics**	**Mean(SD) or %**
Age (years)	28.3 (5.8)
Body Mass Index (kg/m^2^)	23.1 (2.7)
Alcohol consumption (drinks/week)	10.4 (10.2)
Coffee (cups/day)	4.1 (3.5)
Current smokers %	37
Medicine use last month %	10

The men provided a blood sample between December 1993 and March 1994, and for 85 of the participants again in October 1994. Blood samples were collected at the greenhouses in a mobile laboratory. The major reason for drop out was: change of working place, absence from the greenhouse because of having to attend gardening school or closure of the greenhouse. All blood samples were taken between 06.00 and 10.00 AM and after centrifugation serum were frozen at -80°C until analysis. Analysis of TT3, TT4 and TSH was performed at the Central Laboratory at Aarhus University Hospital on an Advia Centaur^®^, (Bayer HealthCare LLC, New York, USA) using competitive immunometric methods [8]. The TSH third generation assay is a sandwich immunoassay using paramagnetic microparticles as solid phase and direct chemiluminescence of acridinium ester for detection of the signal. The assays for TT4 and TT3 are competitive immunoassays with SPALT (solid-phase antigen luminescence technique) architecture using paramagnetic microparticles as solid phase and direct chemiluminescence of acridinium ester for detection of the signal. Analysis of FT3 and FT4 were performed at the Medical Research Laboratory, Aarhus University Hospital using radioimunoassy methods as previously described [9].

The reference values was for TSH: 0.100 to 4.00 mU/l; TT4: 60 to 140 nmol/l; TT3: 1.10 to 2.50 nmol/l; FT4: 12 to 33 pmol/l; and FT3: 3.7 to 9.5 pmol/l. The intra assay coefficient of variance was for TSH: 7%, TT4: 8%, TT3: 6%, FT4: 11% and FT3: 9%.

All analyses of thyroid hormones were performed in 2006.

A comprehensive questionnaire with self-completed information on reproductive, medical, occupational and life style history was checked by a physician. The performance of the questionnaire was tested in repeated pilot surveys among greenhouse workers and the validity of the questions were evaluated by interviews in a subgroup of workers that had filled in the questionnaire. Job tasks were recorded at the start of the study and during a two weeks period in the autumn of 1994. The greenhouse owners, foremen and applicators, provided information on all pesticide applicators from October 1993 to October 1994 (one person per greenhouse provided the information). All participants signed an informed consent and the local ethical committee of Aarhus County approved the study.

### Exposure assessment

More than 60 pesticides were used in the 30 greenhouses included in the study and thyroid disturbing effects of several of these compounds has previously been demonstrated. The compounds used in more than 10% of the greenhouses are presented in Table 2. Thyroid disturbing effects have been demonstrated for the following insecticides: deltamethrin, endosulfan and chlorpyrifos [10-12] and the following fungicides: carbendazim, vinclozolin and thiram [13-15]. Among these, the only demonstration of effects on thyroid hormones among humans was observed in association to the chlorpyrifos metabolite 3,5,6-trichloro-2-pyridinol (TCPY) [12], but the effects of most of the compounds are unknown. Two of the compounds in Table 2 (Vinclozolin and Thiram) have been prohibited for use since the collection of samples for this study.

**Table 2:** Pesticides used in more than 10% of the Greenhouses.

**Pesticide**	**n and (%) of greenhouses**
**Insecticides**	
Pirimicarb	26 (87)
Methomyl	14 (47)
Deltamethrin	14 (47)
Endosulfan	12 (40)
Chlorpyrifos	9 (30)
Buprofezin	9 (30)
Dienochlor	8 (27)
Fenpropathrin	7 (23)
Alphacypermethrin	6 (20)
Amitraz	4 (13)
**Fungicides**	
Benomyl	16 (53)
Iprodione	16 (53)
Chlorothalonil	13 (43)
Carbendazim	8 (27)
Vinclozolin	8 (27)
Thiram	4 (13)
**Growth regulators**	
Chlormequat chloride	23 (77)
Daminozide	17 (57)
Paclobutrazol	12 (40)

From the questionnaire we have several measures of exposure to pesticides.

1) *Spraying load*: Measured at the greenhouse level as the area sprayed in a 3 month period in the summer representing the main spraying period divided by the total greenhouse area.

2) *Spraying hours*: The average number hours actively spraying per year for each individual.

3) *Glove use*: The use of gloves or not during work with culture plants, characterized as always (n = 20), sometimes (n = 48) and never (n = 53).

4) *Exposure years*: The number of years worked in a greenhouse.

5) *Job task*: The greenhouse workers were in addition classified according to job tasks and work practice in three groups taking into consideration all aspects of job function and exposure to evaluate the transfer of pesticides to individuals. This evaluation was performed by 2 agronomists and one occupational health physician. The groups can be characterized as follows: 1 – Low level exposure – working with administration, care and surveillance, pot machine, moving tables and newly hired (n = 44); 2 – medium level exposure – working with packing, care and surveillance, spacing cultures, pricking and potting (n = 45), and 3 – high level exposure – working mainly with nipping and cutting cuttings (n = 13). In addition to job task estimated transfer of pesticides, the amount of pesticides used in the greenhouses were used to estimate the individual exposure supported by measurement of pesticides on gloves from workers during 8 tasks from 3 greenhouses. A more detailed description of the classification can be found in [16].

In the longitudinal study, it was assumed that samples collected in the fall were representing people that had recently been exposed to higher level of exposure than the samples collected in the spring, since the total load of spraying is highest in the summer period just preceding the autumn sample, although pesticide exposure occurs throughout the year for these greenhouse workers, and some groups of pesticides e.g. fungicides were used in a higher volume in the spring [17].

### Statistics

#### Cross-sectional analyses

The difference between exposure groups in thyroid hormone levels in the spring and fall respectively was assessed by analysis of variance. Exposures that could be measured on a continuous scale were dichotomized at the median value to make two exposure groups of equal size. The visual inspection of the distribution of the hormones did not indicate major deviations from normal distributions and therefore no transformation was applied before analysis.

The potential confounding effects of age, body mass index (BMI), alcohol and coffee consumption, smoking and medicine use last month was evaluated by multiple regression analysis with stepwise entering the potential confounders one at the time. None of tested potential confounders changed the estimated mean hormone concentration more than 10% in any of the exposure groups and were therefore not included as confounders in the final model.

For the exposure measures that could be determined on a continuous scale additional regression analysis for the association between the exposure measures (spraying load, spraying hours and exposure years) as independent variables and the thyroid hormones as dependent variables were performed.

#### Longitudinal analysis

The difference in thyroid hormone levels between seasons was assessed by paired t-tests, comparing samples from the same subjects between the spring and the fall.

All statistical analyses were performed using SAS ver. 9.13 (SAS institute, Cary, NC, USA).

## Results

The distribution of thyroid hormones in the spring and fall in different exposure groups is presented in Tables 3, 4, 5.

**Table 3:** TT3 and FT3 hormones at different sampling times and in different exposure groups

	TT3 (nmol/l)		FT3 (pmol/l)	
	Spring n = 121	Fall n = 78	Spring n = 120	Fall n = 86

**Exposure groups**	Mean (95%CI)	Mean (95%CI)	Mean (95%CI)	Mean (95%CI)

Low spraying load < 7.2	2.01 (1.93;2.10)	2.16 (2.01;2.31)	5.81 (5.57;6.05)	6.77 (6.2;7.35)^a^
High spraying load ≥ 7.2	2.08 (2.00;2.17)	2.18 (2.05;2.30)	6.07 (5.83;6.31)	6.28 (5.76;6.80)
Spraying hours pr year < 40	2.03 (1.95;2.10)	2.14 (2.01;2.28)	5.96 (5.73;6.19)	6.47 (5.93;7.01)
Spraying hours pr year ≥ 40	2.04 (1.97;2.12)	2.19 (2.05;2.32)	5.93 (5.71;6.15)	6.53 (5.99;7.07)
Uses gloves always	2.02 (1.88;2.15)	2.21 (1.96;2.45)	6.02 (5.63;6.41)	6.76 (5.81;7.72)
Uses gloves sometimes	2.06 (1.97;2.14)	2.23 (2.07;2.38)	5.90 (5.65;6.15)	6.52 (5.85;7.18)
Uses gloves never	2.04 (1.96;2.12)	2.10 (1.97;2.24)	5.95 (5.70;6.19)	6.40 (5.85;6.95)
Exposure up to 8 years	2.02(1.94;2.10)	2.18 (2.02;2.33)	6.05 (5.82;6.29)	6.39 (5.77;7.00)
Exposure more than 8 years	2.05 (1.97;2.12)	2.16 (2.04;2.28)	5.85 (5.63;6.06)	6.57 (6.08;7.06)^a^
Job task low exposure	1.97 (1.88;2.06)	2.20 (2.06;2.35)^a^	5.79 (5.52;6.05)	6.41 (5.81;7.01)^a^
Job task medium exposure	2.06 (1.98;2.13)	2.14 (2.00;2.27)	6.09 (5.87;6.31)	6.44 (5.88;7.00)
Job task high exposure	2.16 (1.99;2.32)	2.14 (1.82;2.46)	5.78 (5.30;6.26)	6.99 (5.92;8.06)
All	2.04 (1.98;2.09)	2.17 (2.07;2.26)^a^	5.94 (5.78;6.10)	6.50 (6.12;6.87)^a^

a) Difference in the fall sample from the spring sample in a longitudinal analysis (p < 0.05).

**Table 4:** TT4 and FT4 hormones at different sampling times and in different exposure groups

	TT4 (nmol/l)		FT4 (pmol/l)	
	Spring n = 121	Fall n = 78	Spring n = 120	Fall n = 86

**Exposure groups.**	Mean (95%CI)	Mean (95%CI)	Mean (95%CI)	Mean (95%CI)

Low spraying load < 7.2	93.4 (89.7;97.1)	93.5 (88.8;98.2)	23.1 (21.8;24.4)	26.4 (24.2;28.7)^a^
High spraying load ≥ 7.2	94.4 (90.8;98.0)	96.4 (92.5;100.3)	20.7 (19.5;22.0)^b^	22.1 (20.0;24.2)^b^
Spraying hours pr. year < 40	94.0 (90.4;97.7)	93.8 (89.6;98.0)	22.4 (21.1;23.7)	24.6 (22.4;26.8)^a^
Spraying pr year ≥ 40	92.7 (89.1;96.2)	96.9 (92.8;101.0)	21.8 (20.6;23.1)	23.7 (21.5;25.9)
Uses gloves always	92.2 (85.7;98.6)	93.5 (85.8;101.2)	21.4 (19.1;23.6)	23.5 (19.6;27.4)
Uses gloves sometimes	93.2 (89.1;97.2)	94.7 (89.7;99.7)	21.5 (20.0;22.9)	23.2 (20.5; 25.9)
Uses gloves never	94.1 (90.3;98.0)	96.5 (92.1;100.8)	23.0 (21.6;24.4)	25.0 (22.8;27.2)^a^
Exposure up to 8 years	91.0 (87.3;94.7)	93.7 (88.9;98.5)	21.2 (19.9;22.6)	22.8 (20.4;25.3)
Exposure more than 8 years	95.4 (92.0;98.9)	96.5 (92.7;100.2)	22.9 (21.7;24.1)	24.9 (23.0;26.9)^a^
Job task low exposure	91.3 (87.1;95.5)	96.6 (92.1;101.2)	21.3 (19.7;22.8)	22.9 (20.5;25.3)^a^
Job task medium exposure	93.5 (90.1;97.0)	95.5 (91.3;99.7)	22.7 (21.4;24.0)	24.3 (22.0;26.5)
Job task high exposure	99.2 (91.5;106.9)	89.1 (79.3;99.0)	22.2 (19.5;25.0)	27.6 (23.3;31.9)
All	93.3 (90.8;95.9)	95.4 (92.4;98.4)	22.1 (21.2;23.0)	24.1 (22.6;25.7)^a^

a) Difference in the fall sample from the spring sample in longitudinal analysis (p < 0.05).

b) Difference from the low exposure group in cross-sectional analysis (p < 0.05).

**Table 5:** TSH hormone at different sampling times and in different exposure groups

	TSH (mU/l)	
	Spring n = 119	Fall n = 76

**Exposure groups.**	Mean (95%CI)	Mean (95%CI)

Low spraying load < 7.2	1.86 (1.63;2.10)	1.33 (1.10;1.56)^a^
High spraying load ≥ 7.2	1.70 (1.47;1.92)	1.43 (1.24;1.62)^a^
Spraying hours pr. year < 40	1.95 (1.69;2.22)	1.50 (1.29;1.71)^a^
Spraying pr year ≥ 40	1.72 (1.45;1.98)	1.29 (1.10;1.49)^a^
Uses gloves always	1.76 (1.28;2.23)	1.52 (1.15;1.89)
Uses gloves sometimes	1.90 (1.60;2.21)	1.33 (1.08;1.57)^a^
Uses gloves never	1.81 (1.52;2.10)	1.40 (1.19;1.61)^a^
Exposure up to 8 years	1.78 (1.51;2.06)	1.55 (1.31;1.78)
Exposure more than 8 years	1.88 (1.62;2.14)	1.30 (1.12;1.48)^a^
Job task low exposure	1.82 (1.51;2.13)	1.51 (1.19;1.73)^a^
Job task medium exposure	1.75 (1.49;2.01)	1.26 (1.06;1.46)^a^
Job task high exposure	2.27 (1.70;2.84)	1.59 (1.08;2.10)
All	1.83 (1.65;2.02)	1.39 (1.25;1.54)^a^

a) Difference in the fall sample from the spring sample in longitudinal analysis (p < 0.05).

### Cross sectional analyses

FT4 was lower among the persons working in greenhouses with a high spraying load in the spring and in the fall (Table 4). In addition, when analyzed on a continuous scale FT4 decreased by -0.25 pmol/l CI (-0.41;-0.08) per unit increase in spraying load (indicating per time the total greenhouse area is sprayed) in the spring and -0.29 pmol/l CI (-0.59;0.01) in the fall. None of the other cross-sectional analysis revealed statistical significant differences between exposure groups in thyroid hormones.

### Longitudinal analyses

For TT3 and FT3, an overall increase from the spring to the fall sample was observed (Table 3). When divided into exposure groups, the increase of FT3 was most pronounced among people working in greenhouses with a lower than median spraying load, for the persons working for more than 8 years in a greenhouse and for the persons classified as low exposure based on job tasks. Also for TT3 the difference between seasons was most pronounced for persons classified as low exposure based on job tasks.

A significant increase in FT4 but not TT4 level was observed from the spring to the fall (Table 4), this increase being most pronounced among workers from greenhouses with a low spraying load, among persons with low number of spraying hours, among greenhouse workers never using gloves, among those exposed for more than 8 years to the greenhouse environment, and among those classified as low level exposure.

The strongest differences between spring and fall were seen in TSH, with a markedly lower level in the fall.

The difference between seasons was statistical significant in all of the high exposure groups except for job task classification, and also for several of the low exposure groups, but not among gardeners always using gloves and among those working for less than 8 years in a greenhouse.

### Evaluation of elevated or decreased level of thyroid hormones

For in total 24 individuals one or more of the thyroid hormone measurements was out of the reference range [see Additional file 1]. For most individuals the difference was only slightly out of the reference range and for only 4 persons the measurements was consistently out of range in both the spring and fall measurement. In the spring increased TSH was observed in 5 persons whereas none showed elevated TSH in the fall. Increased TT3 was the most commonly observed deviation, with 8 subjects showing elevated TT3 in the spring – 3 of these still had elevated TT3 in the fall – and additionally 7 individuals not showing elevated TT3 in the spring had elevated TT3 in the fall. It is worth to note that the level of TT3 was below the action level in all cases. Exclusion of subjects outside of the reference range in both spring and fall did not change the level of significance of the findings in the analyses of differences between exposure groups.

## Discussion

The level of FT4 was decreased among persons working in greenhouses with a high spraying load both in the spring and in the fall. Furthermore a shift towards lower level in TSH and a higher level of FT3, TT3, and FT4 from the spring to the fall was observed.

The consistently lower FT4 level among people working in greenhouses with high exposure to pesticides suggests that this decrease may be related to pesticide exposure. Thyroid disturbing effect after pesticide exposure with effects on FT4 has previous been observed among pesticide applicators from the Red River Valley of Minnesota, [4]. Also animal studies support that pesticide exposure causes a decrease in thyroid hormones, but often the decrease in TT4, FT4, TT3 and FT3 is accompanied by an increase in TSH, which was not observed in the present study [5].

In the spring in general a higher TSH and lower TT3, FT3 and TT4 level was observed compared to the fall. In this population it has previously been demonstrated that the level of pesticide exposure is highest in the summer and fall period [17]. Therefore, we would expect the opposite of the observed (lower level of TT3, FT3, TT4 and FT4 and higher TSH) in the fall, if it was caused by pesticide exposure in general. However, specific pesticides used in the spring and not in the fall e.g. fungicides may have a more harmful effect on thyroid function than the pesticides used in the summer and fall. In a series of American studies on pesticide applicators from the Red River Valley, fungicides seemed to be the most harmful group of pesticides to thyroid disrupting effects [18].

In our study, the analysis stratified on exposure groups revealed that about the same number of groups classified as high and low exposure showed statistical significance comparing spring and fall samples, and therefore no clear association to toxicant exposure in the seasonal differences can be determined.

It must be considered that the blood samples were from a period without iodine supplementation of table salt in Denmark, which in itself may cause a moderate hypothyroid status. Based on the information in a large study of thyroid function in the Danish population, approximately 1% of younger men should have sub-clinical hypothyroidism, and less than 0.5% should have clinical hypothyroidism [19]. Due to the relative high incidence of thyroid diseases it was decided to start iodine supplementation of table salt in 1998 in Denmark. Dietary changes from the spring to the fall may explain the general shift in thyroid hormone values, since the thyroid status of this moderately iodine depleted population was probably highly sensitive to iodine contents in the food. Furthermore, previous studies have demonstrated seasonal variation thyroid hormones, similar to the effects in the present study, in populations without known pesticide exposure [20-22] although the seasonal variations in thyroid hormones is not entirely consistent between studies.

One limitation with the present study is the lack of exact exposure measurements. The exposure was estimated as average exposure based on spraying activities and work tasks. It has previously been estimated that greenhouse workers absorb the major part of the pesticides through handling of cultures that has been sprayed with pesticides, and therefore both spraying load and work tasks in a greenhouse is important for the actual exposure of the individual [6]. In the present study we did only find associations between spraying load and thyroid hormones.

Previous studies on the same population have indicated that semen quality was decreased among the most highly exposed men based on job tasks and for the men working for several years with exposure to pesticides [16]. Furthermore, a seasonal difference in chromosome aberrations was found with more chromosome aberrations in the fall (after the spraying season), and mostly so among the persons who did not use gloves during re-entry activities [17].

Since spermatoxic, genotoxic and thyroid disturbing effects are not necessarily caused by the same pesticides, the discrepancy in the exposure groups being mostly affected in the previous and the present study may be explained by unequal exposure of individuals to the single pesticides causing spermatoxic, genotoxic and thyroid disturbing effects.

Since the greenhouse workers are exposed to a number of chemical compounds, we cannot with the present study pinpoint which chemicals that are the culprits for the observed effects. Several compounds has not been tested for their effects on thyroid function, and therefore we can not simply determine which of the used chemicals that are likely to cause the observed effects. However, several of the pesticides used (Deltamethrin, Chlorpyrifos and Vinclozolin) have been associated to a decrease in FT4 level in previous studies [10,12,14].

The dichotomizing of the gardeners into low and high exposed groups may not give an appropriate exposure contrast since all greenhouse worker involved in the present study are to some extend exposed to pesticides. Therefore, it can be argued that an external control group should be used. However, when using the low level exposed gardeners as a control group, the high and low level exposed group are more likely to be similar on several other factors that may confound the results.

Furthermore, dichotomizing of exposure may underestimate the effects of pesticides on exposure. However, when we analyzed the data that could be regarded as continuous in a linear regression model the same associations turned out as being statistically significant, indicating that at least these associations was not simply based on the choice of exposure groups.

Finally, the sample size in some of the groupings is quite low. Especially the number of individuals classified as high-level exposure based on job tasks (n = 13) provides limited power. However, we believe the different approaches to exposure estimation would reveal effects of pesticides on thyroid hormones among greenhouse workers, if any. It should, however, be acknowledged that type and level of exposure might be much higher in other settings with less stringent exposure control than in Denmark.

## Conclusion

Thyroid function was only marginally different among Danish greenhouse workers with different levels of exposure to pesticides. The only difference observed was a moderate reduction in FT4 level among the greenhouse workers working in greenhouses where the spraying load were high. A seasonal difference in thyroid function was observed, but it was not more pronounced in the high exposure groups compared to low exposure groups, and therefore other causes than pesticide exposure may explain the differences.

## Abbreviations

BMI Body mass index (kg/m^2^)

EDBC Ethylenebis(dithiocarbamate)

FT3 Free triiodothyroxine

FT4 Free thyroxine

TSH Thyroid stimulating hormone

TT3 Total triiodothyroxine

TT4 Total thyroxine

## Competing interests

The author(s) declare that they have no competing interests.

## Authors' contributions

JP was responsible for collection on serum samples and AF was responsible for analysis of FT3 and FT4. GT coordinated the study and drafted the manuscript. All authors participated in the design of the study, commented on the draft and have read and approved the final manuscript.

## Supplementary Material

Additional file 1 List of subjects with at least one thyroid hormone values out of reference range. The reference range is indicated in the first row. Entries marked in bold indicate values out of reference range. The file contains a table listing individual hormone measurements for the individuals with at least one thyroid measurement out of reference range. Click here for file
