# Supplementary material for: Thyroid function in Danish greenhouse workers
Source: Environ Health. 2006 Dec 6;5:32. doi: 10.1186/1476-069X-5-32 (PMC1698912; doi:10.1186/1476-069X-5-32)
Supplement: Additional file 1 — List of subjects with at least one thyroid hormone values out of reference range. The reference range is indicated in the first row. Entries marked in bold indicate values out of reference range. The file contains a table listing individual hormone measurements for the individuals with at least one thyroid measurement out of reference range. [file 1476-069X-5-32-S1.doc]

| **Subject nr** | **TSH spring**  **0.1-4.0 mU/l** | **TT3 spring**  **1.1-2.5 nmol/l** | **TT4 spring**  **60-140 nmol/l** | **FT3 spring**  **3.7-9.5 pmol/l** | **FT4 spring**  **12-33 pmol/l** | **TSH fall**  **0.1-4.0 mU/l** | **TT3 fall**  **1.1-2.5 nmol/l** | **TT4 fall**  **60-140 nmol/l** | **FT3 fall**  **3.7-9.5 pmol/l** | **FT4 fall**  **12-33 pmol/l** |
| --- | --- | --- | --- | --- | --- | --- | --- | --- | --- | --- |
| 1 | 1.46 | 2.14 | 94 | 5.9 | **34** |  |  |  |  |  |
| 2 | 0.93 | **2.59** | 120 | 5.9 | 21 |  |  |  |  |  |
| 3 | 2.03 | **3.06** | 99 | 5.6 | 15 | 1.27 | **3.31** | 103 | 6.80 | 14 |
| 4 | 1.86 | **2.55** | 94 | 4.9 | 16 | 2.17 | **3.04** | 124 | 6.80 | 20 |
| 5 | **4.16** | 2.26 | 112 | 5.8 | 21 | 2.51 | **2.53** | 116 | 5.6 | 23 |
| 6 | 2.15 | 2.11 | 88 | 4.7 | 19 | 1.09 | **2.68** | 124 | 6.30 | 22 |
| 7 | 3.46 | 1.80 | 80 | 4.7 | 18 |  | **4.31** | 102 | **12.8** | 28 |
| 8 | 1.39 | 1.28 | 94 | 4.4 | **41** |  |  |  | **16.1** | **62** |
| 9 | 1.33 | **1.09** | 82 | 6.4 | 23 | 1.19 | 2.18 | 99 | 6.6 | 24 |
| 10 | **6.98** | 1.94 | 109 | 6.1 | 21 | 1.99 | 1.87 | 109 | 6.2 | 25 |
| 11 | 2.54 | **2.51** | 121 | 7.1 | 30 | 0.82 | 2.11 | 97 | 6.50 | 29 |
| 12 | **6.14** | 1.90 | 63 | 7.2 | 13 |  |  |  |  |  |
| 13 | 0.74 | 1.69 | 91 | 4.4 | 20 |  |  |  | **16.1** | **60** |
| 14 | 0.95 | 2.18 | 107 | 6.6 | 21 | 0.88 | **2.60** | 109 | 6.80 | 29 |
| 15 | **4.36** | 2.26 | 106 | 6.4 | 19 | 2.62 | 1.97 | 90 | 5.4 | 21 |
| 16 | 1.57 | **2.55** | 110 | 7.6 | 28 | 0.70 | 2.40 | 98 | 5.20 | 22 |
| 17 | 0.74 | 2.42 | 103 | 7.0 | 27 | 0.77 | **2.64** | 109 | 6.80 | 29 |
| 18 | 1.43 | 1.97 | 106 | 5.6 | 21 | 0.87 | **2.88** | 106 | 7.80 | 30 |
| 19 | 2.36 | 2.17 | 94 | 6.1 | 20 | 1.55 | 1.97 | 86 | 6.30 | **37** |
| 20 | 0.89 | 2.19 | 75 | **10.8** | 20 |  |  |  | 6.6 | 16 |
| 21 | 2.22 | 2.28 | 85 | 5.9 | 24 | 1.81 | **2.57** | 104 | 7.10 | 17 |
| 22 | 3.00 | **2.60** | 130 | 6.9 | 28 | 1.77 | **2.63** | 126 | 5.80 | 16 |
| 23 | **6.02** | **3.16** | 123 | 6.9 | 21 |  |  |  |  |  |
| 24 | 1.10 | 2.02 | 90 | 5.8 | 18 | 0.75 | **2.51** | 98 | 7.50 | 24 |

**Additional file 1.** List of subjects with at least one thyroid hormone values out of reference range. The reference range is indicated in the first row. Entries marked in bold indicate values out of reference range.
